# Medical Opinion and Movement

**Published:** 1908-12-26

**Authors:** 


### Medical Opinion and Movement.

THE operative treatment of fractured patella and several other surgical conditions of the knee-joint form the subject of a paper by Dr. Tenney in the *Annals of Surgery*. The most interesting of his contentions with reference to the former is that he advocates the use of absorbable suture material (chromicised catgut) rather than that of wire. It appears that at the present day in two representative American hospitals absorbable sutures are used for this operation thrice as often as non-absorbable. Careful suture of the aponeurosis is also regarded as an integral part of the operation. The author accounts for the much greater difficulty of maintaining asepsis in knee-joint operations than in laparotomies partly by the different qualities of the skin and partly by the constant motility of the abdominal parietes. The peritoneum, he points out, is never at rest, and in consequence the natural drainage by stomata is given full play: whereas in a knee-joint treated by the usual immobilisation the opportunities of such drainage are reduced to a minimum. Not only is he convinced that complete immobilisation is bad for the prophylaxis of sepsis in the after-treatment of opened knees, but also that it is inadvisable when the joint is definitely septic. For the same reason he favours especially free artificial drainage in all operations in which the joint is opened. Dr. Tenney does not believe that a septic knee-joint must necessarily end in ankylosis, if drainage is sufficiently free and a sufficient amount of movement insisted on. He also lays stress upon the advantages of full flexion of the knee during the pre-operative cleansing of the skin, to flatten out the small crinkles which furrow the skin during extension. Among the other topics discussed is the treatment of prepatellar bursa by incision and drainage under cocaine, after which the patient can at once resume work (other than kneeling). Internal derangements are also dealt with; these he thinks less often due to injury of the semi-lunar cartilage than to ligamentous damage, and in severe cases where lateral and torsion movements are too free he would perform primary suture of the ruptured ligament.

WHEN it was scientifically established that the rat-flea can and does transfer the bacillus pestis from infected rats to non-infected human beings, not unnaturally the eradication of rats in plague districts became a matter of extreme urgency. But unless it is the case that plague can be transferred to man in no other way than *via* the infected rat and its infected parasite, it is quite obvious that the precautions usual to prevent the spread of a highly infective disease cannot be abandoned altogether in favour of rat destruction. According to the *Journal of Tropical Medicine and Hygiene*, not only is this proposition not proved, but it has even been quite disproved by strict inquiry made by experts of high standing. Certainly there is point also in the contention that if plague can only be spread by the rat-flea method the active campaign against these rodents and their parasites should have produced better results by this time. We gather that the Plague Research Commission has been concentrating its prophylactic energies upon pulicides, such as kerosene oil emulsion, and has displayed some indifference towards the disinfection of fæces by antiseptics: such a policy calls forth the strong criticism of the *Journal*. Whatever the real truth of the matter as concerns the dissemination of plague may turn out to be, it must be agreed that in the present state of affairs "it is impossible, with the conflicting opinions of so many capable observers, for the interested bystander to arrive at a final conclusion."

CHAUFFARD has recently communicated to the Paris Academy of Medicine a monograph upon primary carcinoma of the body of the pancreas, which differs essentially from cancer of the head. In the latter there is seldom pain, and the characteristic symptom is chronic progressive jaundice without intermittence, accompanied by marked and constant dilatation of the gall bladder. In cancer of the body of the pancreas, which is considerably rarer, jaundice only appears at a late stage, if at all, and there is no dilatation of the gall bladder. The early appearance of paroxysmal pain, situated first to the left and shifting towards the middle line, accompanied by frequent false calls to stool, are characteristic symptoms. The pains are constant and excruciating. They may radiate towards the scapula and spine. Patients only obtain relief when seated and leaning forwards. Loss of flesh and cachexia rapidly follow, but there is no glycosuria. The pains are like the girdle pains of tabes, and their presence can be explained by the close relation of the body of the pancreas with the semilunar ganglia and solar plexus. Relief from pain can be effected by early laparotomy and freeing of the adhesions round the abdominal viscera and nerves.

DIURETIC drugs form the subject of a communication by Professor Romberg to the *Münchener Medizinsche Wochenschrift*. Diuresis can be effected in two ways. Firstly by acting reflexly on the kidneys by drugs which improve the general circulation and hence the blood supply of the organs; secondly by drugs acting directly upon the kidney substance. It was thought that the circulation could be improved by raising the blood-pressure, but it has been shown that drugs which increase blood-pressure do not always promote diuresis, and moreover often have a specific action of their own which hinders it. It is necessary not only to improve the tonus of the heart, but that of the whole circulatory system as well, for œdema often only disappears after the obstacle to the peripheral circulation has been removed. Thus, ascites disturbs the circulation of the lower limbs and the kidneys by pressure on the veins draining these parts. Diuresis can only be established, in such cases, after evacuation of the ascitic fluid. Drugs which act directly on the renal substance are especially useful in draining the organism of fluid. Recourse should be had to these in cases of cardiac insufficiency where tonic drugs have failed to act upon the heart. The author believes that the best diuretics are to be found among those of the purin series. These act directly on the kidney and only fail when there is some lesion present of the kidney vessels, especially the glomeruli.

---

THE most active drug of this series is dimethylxanthin, otherwise known as theophyllin or theocine. This should first be given in .1 gram doses twice a day, and gradually increased to .2 gram three or four times daily. Diuretin is less active, theobromine still less so, caffeine coming last of all. The vegetable diuretics, though useful, are less potent. Such are preparations of juniper berries, asparagus, birch, etc. Calomel and sodium salicylate have a distinctly injurious action on the kidney. Salts, milk-sugar (recommended by Sée), and copious draughts of water are of little use in stimulating a flagging kidney to increased secretion. Potassium acetate, if given in doses of twenty to thirty grams daily, is more efficacious. The only diuretics of use, however, in cases in which there is renal disease, or much circulatory disturbance, are theocine and diuretin. When prescribing these drugs the intake of water should be reduced to three pints or so in the twenty-four hours. A chloride-free diet improves the patient's condition in cases of dropsy and uræmia of renal origin, but care should be taken not to go too far in prohibiting salt. The author has been able to show that in cases of ascites due to tuberculous peritonitis a salt-free diet increases diuresis and hastens the dispersal of collections of ascitic fluid.

---

RAOUL LABBE discusses in *La Revue de Gynécologie et d'Obstétrique*, the prognosis, immediate and remote, of infantile convulsions. Among the commoner remote consequences are transitory and permanent contractures, strabismus, transitory hemiplegias, and stammering. It is assumed that these are for the most part due to cerebral œdema or effusions of blood at the moment of onset of the convulsions. Sometimes, as is well known, convulsions are the first manifestations of epilepsy, for, according to different authorities, from seven to 30 per cent. of such children eventually become epileptics. On the other hand only about 10 per cent. of epileptics have their first fit before the age of three. Apart from the above-mentioned consequences, convulsions sometimes lead to a progressive mental deficiency ending in idiocy. Different statistics have attempted to explain the nature of the onset of infantile convulsions. Out of 91 new-born infants seized with convulsions, Bouchut classified 34 as suffering from "symptomatic" convulsions and 57 from "essential" convulsions; of these last 25 were afterwards completely cured, 11 were lost to view, and the rest died from various causes. More recently Porfeschnigg, out of 109 cases, reported a mortality of 72 per cent. Of those who left hospital alive, 52.6 per cent. died before puberty. Thus, according to this authority, prognosis is always very grave in cases of infantile convulsions.

TWO cases of tumour of the middle fossa of the skull are reported by Dr. Judson Bury in the *Medical Chronicle*. The first was that of a bricksetter who for eighteen months before admission to hospital suffered from neuralgic pain in the right eye and right side of the face. For six months this had been getting worse, and vision in that eye was eventually lost. There were no symptoms referred to any of the limbs, and the diagnosis was established from the combination of severe pain and anæsthesia of the right side of the face, with neuro-paralytic ophthalmia and ophthalmoplegia. The evidence showed that the third, fourth, fifth, and sixth nerves were alone affected, and this syndrome suggested a tumour of the middle fossa. Subsequently the *left* seventh nerve became involved also, a short time before death. The evidence of the lesion of the right trigeminal was afforded not only by pain and anæsthesia, but also by neuropathic keratitis and by swelling and ulceration of the right side of the palate and buccal cavity. Postmortem a growth about the size of a small orange was found in the right middle fossa, involving the Gasserian ganglion and extending forwards into the orbit; microscopically the tumour was an endothelioma. In the other case the clinical history was very similar; for sixteen months the symptoms were purely subjective, chiefly severe and constant headache made worse by movement and noise. During the remaining three months of life there were definite physical signs of implication of the third, fourth, fifth, sixth, and eighth nerves on the right side, and of the seventh and twelfth on the left. As in the other case, the limbs were unaffected, and there was neither optic neuritis nor vomiting throughout. Curiously, just before death there was an apparent improvement: the deafness diminished, ptosis almost disappeared, and subjective sensations were less marked. The tumour was an endothelioma of the right middle fossa.

---

AN interesting discussion upon the treatment of pernicious anæmia took place at a recent meeting of the Berlin Society of Medicine. Drs. Morgenroth and Reicher claim to have obtained successful results in the treatment of this disease by the administration of cholesterin. The treatment is based on the assumption that the anæmia is due to certain hæmolysines in the blood of the patients, and that cholesterin acts as an antihæmolytic. They recommend a dose of 3 grams of cholesterin dissolved in 100 grams of olive oil per diem. Dr. Klemperer rejects the theoretic assumptions on which the treatment is based on the grounds that the presence of hæmolysines in the blood of patients affected with the disease has not been demonstrated. He does not, however, on that account reject the treatment, but points out that it can be carried out in a more agreeable form by prescribing a diet with a large proportion of butter and cream, both rich in cholesterin. He orders 100 to 200 grams of butter (about ¼ to ½ lb.) and a half to one litre of cream per diem. He has obtained very satisfactory results with this diet, the number of red corpuscles increasing rapidly, together with considerable increase in body-weight.

But he naturally suggests that the beneficent effect may be due rather to the increased nourishment than to the contained cholesterin. He has seen the best results by combining this diet with the administration of arsenic. He favours especially injections of arsacetine, which is supposed to be six times more active than atoxyl, and has been used by Ehrlich in trypanosomiasis. Injections of 60 centigrams of arsacetine are made on two successive days, followed by an interval of five days, and this course of treatment is continued for a month. It is stated, however, that this method is liable to produce symptoms of dyspepsia and fever, especially in women; but the improvement in the blood condition has been very marked in several cases.

---

MECHANO-MORPHOSIS, or the influence of the functional factors on the processes of repair of the tissues: such is the subject of a highly interesting paper by Dr. G. Fichera in the "Archives of Experimental Medicine and Pathological Anatomy." The author has studied in the dog the microscopic changes which take place during the reparative processes after traumatic injury of certain tissues—namely, the pleura, the lungs, striated muscles and blood-vessels. In the case of the muscles it is already known that the nutrition of the muscles depends upon the integrity of the blood-vessels on the one hand, and of the nervous system on the other, and that division of the motor nerve leads to atrophy of the muscular fibres thus deprived of their innervation. The author is led, however, by his researches to deny any supposed trophic influence exercised by the motor nerve upon the muscular fibres. According to him, the atrophy is due to the inactivity of the muscle, and the same degenerative changes are to be observed when the contractions of the muscle are abolished by tenotomy.

---

IN regard to repair after section of the muscle, the author finds also a marked difference according as the muscle is left in a state of functional activity or rendered immobile by division of the tendon or the motor nerve. In the latter case degeneration of the divided fibres is more extensive and more lasting, reparation is slow and incomplete, and the formation of new fibres is sparse. On the other hand, if the movements of the muscle are preserved, the divided fibres degenerate very little and heal rapidly. Moreover, the more often the muscle is allowed to contract, the better and more quickly does it heal. For instance, it is stated that the most complete repair is obtained in the diaphragm muscle, of which the continual rhythmic contractions are necessary to life. A study of the reparative process of the injury to the blood-vessels shows that the same principles rule here also. Repair of the injured vessel is much more rapid and complete if the vessel remains permeable to the blood-stream than if it becomes blocked by a thrombus. It would appear, therefore, from these researches that the therapeutic principle of absolute rest to an injured organ or tissue may be carried too far, and that the exercise of the normal function of the tissue in a moderate degree will best further its complete restitution. This fact has already been grasped to a large extent in the present day treatment of fractures and injured joints, and has given rise to the modern methods of early massage and passive movements of the affected limbs in place of complete and prolonged immobility in plaster of Paris and splints.

---

AN important paper by Dr. J. Lehrmitte has recently appeared in *La Semaine Médicale* on associated movements, or what he terms "complementary opposition," as a means of differential diagnosis between functional and organic paralysis. The author points out that in any voluntary movement, in addition to the contraction of the particular muscle or group of muscles involved in the movement, there are certain secondary phenomena, such as the relaxation of antagonistic muscles and the contraction of other muscles for the fixation of certain parts of the skeleton. When a healthy individual is placed in a horizontal position and is told to raise, say, the right leg, it will be found that the sacro-lumbar muscles contract, and at the same time the left leg is pressed against the bed. This movement of "complementary opposition" is still more marked if resistance is offered to the elevation of the leg. In this case the foot forms a furrow in the mattress from the pressure it exerts. This complementary opposition can easily be verified by placing the hand under the foot, when the pressure will be felt. Conversely, if the patient's right leg is pressed hard against the bed his left leg will be found to be raised slightly at the same time that the pressure is made by the observer.

---

IN cases of hemiplegia or monoplegia of the lower limb of organic origin this phenomenon of opposition on the part of the healthy leg never fails when the patient tries to raise the paralysed leg, and the pressure of the paralysed limb against the bed is also obtained when the opposite limb is raised against resistance. Naturally this counter-pressure of the paralysed side is proportional to the muscular force which may still persist in the muscles affected. It is claimed also that the same phenomena of opposition may be demonstrated in the upper limbs in hemiplegia, although in certain cases it is absent. In functional or hysterical paralysis, on the other hand, the signs of opposition are different. If the patient is told to raise the paralysed limb there is no "movement of opposition" of the healthy leg; but if the healthy leg is raised against resistance, the muscles of the paralysed leg are felt to contract, and the leg is pressed against the bed as in a normal individual. Professor Hoover, of Cleveland, and Professor Zenner, of Cincinatti, have published several interesting cases in which they have been able to demonstrate the functional nature of a hemiplegia by means of these signs. In some cases of organic paralysis the sign of opposition on the paralysed side is accompanied or replaced by some other associated movement, which may take the form of retraction of the limb, with marked flexion at the hip and at the knee.

